# The Use of Machine Learning for Inferencing the Effectiveness of a Rehabilitation Program for Orthopedic and Neurological Patients

**DOI:** 10.3390/ijerph20085575

**Published:** 2023-04-19

**Authors:** Valter Santilli, Massimiliano Mangone, Anxhelo Diko, Federica Alviti, Andrea Bernetti, Francesco Agostini, Laura Palagi, Marila Servidio, Marco Paoloni, Michela Goffredo, Francesco Infarinato, Sanaz Pournajaf, Marco Franceschini, Massimo Fini, Carlo Damiani

**Affiliations:** 1 Department of Anatomy, Histology, Forensic Medicine and Orthopedics, Sapienza University, Piazzale Aldo Moro 5, 00185 Rome, Italy; 2 Department of Neurological and Rehabilitation Science, IRCCS San Raffaele Roma, Via della Pisana 235, 00163 Rome, Italy; 3 Department of Computer, Control and Management Engineering Antonio Ruberti, Sapienza University, Piazzale Aldo Moro 5, 00185 Rome, Italy; 4 Department of Human Sciences and Promotion of Quality of Life, San Raffaele University, Via di Val Cannuta 247, 00166 Rome, Italy

**Keywords:** artificial intelligence, machine learning, rehabilitation, Barthel Index, algorithms, functional improvement

## Abstract

Advance assessment of the potential functional improvement of patients undergoing a rehabilitation program is crucial in developing precision medicine tools and patient-oriented rehabilitation programs, as well as in better allocating resources in hospitals. In this work, we propose a novel approach to this problem using machine learning algorithms focused on assessing the modified Barthel index (mBI) as an indicator of functional ability. We build four tree-based ensemble machine learning models and train them on a private training cohort of orthopedic (OP) and neurological (NP) hospital discharges. Moreover, we evaluate the models using a validation set for each category of patients using root mean squared error (RMSE) as an absolute error indicator between the predicted mBI and the actual values. The best results obtained from the study are an RMSE of 6.58 for OP patients and 8.66 for NP patients, which shows the potential of artificial intelligence in predicting the functional improvement of patients undergoing rehabilitation.

## 1. Introduction

One out of six people in the European Union needs to be referred to rehabilitation services because of the occurrence of a disability, caused by either acute or chronic disease [1]. Once the treatment starts, the evaluation of clinical progress is of importance in all the fields of medicine, including rehabilitation, and it appears critical for determining the effectiveness and efficiency of the selected treatments. Given the nature of disabilities, impacting several domains of persons’ lives differently and often being difficult to objectively measure as a clinical end-point, studies have focused on specific indicators to predict the effectiveness and efficiency of rehabilitation interventions. From this point of view, Rehabilitation Effectiveness (REs) [2,3] and Rehabilitation Efficiency (REy) [4] have been used to measure the results of rehabilitative interventions in different categories of patients [5]. Recent studies [6,7,8] found a trade-off relation between REs and REy concerning the length of stay (LOS) and patient-baseline modified Barthel Index (mBI) [9,10,11]. With a view to the intended use of resources, as well as their best possible use in healthcare, trying to predict the potential functional improvement of patients undergoing rehabilitation in terms of effectiveness and efficiency may help in developing precision medicine tools and tailored, patient-specific rehabilitation [7]. Instead of using statistical methods to identify those factors that may affect rehabilitation processes and their outcomes [7,12,13], recently, machine learning methods have been used [14]. Machine learning is a multidisciplinary field used in many application domains such as computer vision, natural language processing, and medical domains, from images to structured and semi-structured data, which studies algorithms that automatically improve from experience. It is often considered a subfield of artificial intelligence which builds mathematical models and learns generalization patterns from data which are then used to make predictions on unobserved instances [15,16,17,18,19,20]. Because of their flexible nature, machine learning methods can be more accurate than conventional regression or correlation in predicting future scenarios [21,22,23]. Based on this assumption, the present study aims to make advance assessment of the potential functional improvement of post-acute patients undergoing rehabilitation to develop precision medicine tools and patient-oriented rehabilitation programs, as well as to allocate resources in hospital better, using a predictive model in terms of mBI exploiting machine learning algorithms. Specifically, our proposal involves utilizing tree-based ensemble machine learning models, such as xGBT, LightGBM, CatBoost, and gradient boosting [24,25,26], to analyze patient data obtained from the Acceptance/Discharge Report (ADR) [27] during the time of admission to a rehabilitation program. These models can extract complex nonlinear relationships that can accurately model the distribution of rehabilitation outcomes. We chose these models based on their ability to learn from various types of input data, including categorical and continuous data, which is advantageous for our study’s private dataset that consists of multiple variables of different types. In conclusion, this study’s contributions are as follows:

1. To the best of our knowledge, this is the first study that attempts to predict functional improvement from ADR data which represents real-world scenarios registered in Italy.
2. We study the applicability of machine learning in assessing the rehabilitation outcome in advance.
3. An in-depth analysis of how different models and combination affects the accuracy with which the proposed algorithms predict the target variable.

## 2. Materials and Methods

### 2.1. Dataset

We retrospectively evaluated data collected from 2015 to 2018, using a database of approximately 4050 unique hospital discharges at IRCCS San Raffaele of Rome, Italy from the neurology and orthopedy departments referring to records registered in the “Acceptance/Discharge Report for the rehabilitation area” (ADR), which implements the Italian law (DGR 731/2005) [27]. The inclusion criteria for the analysis were: age ≥ 18 years, and time between the onset of the disease and rehabilitation hospitalization ≤ 60 days since we included only post-acute patients defined according to the appropriateness criteria for admission to rehabilitation defined in Italian national laws. In addition, the length of hospitalization was >14 days and ≤90 days from the first day of hospitalization since patients hospitalized for more than 90 days are extremely rare, whereas patients in rehabilitation for less than two weeks are also rare cases of patients in good condition. Moreover, patients with missing data such as hospitalization pathology, age, or functional ability at the time of hospitalization were excluded due to their importance in the rehabilitation process and its outcome. However, the process of treating the missing data is better explained in the Section 2.2. The initial dataset contained 120 items that corresponded to the data items present in the ADR regarding the patients. To protect the privacy and comply with regulations, all data were anonymized by removing identifying information such as names, birth dates, and identity numbers, and assigning a unique random ID. Pathologies were categorized using the standard International Classification of Diseases, Ninth Revision, Clinical Modification (ICD9-CM), and patients were classified into two main groups based on ICD9-CM codes [27]: orthopedic patients (OP) and neurological patients (NP). The reason behind grouping the data in these two macro-categories and not finer categories is due to the small quantity of the data and the intra-group variability in pathologies. In addition, demographic information about the dataset is presented in Table 1, and the range of ICD9-CM macro-categories used to categorize the data is shown in Table 2. The dataset contained mostly categorical variables, with only a few quantitative ones. The study protocol was reviewed and approved by the Ethical Committee of the IRCCS San Raffaele Pisana of Rome on 18/07/2018 (code number 07/18) and was developed in accordance with STROBE guidelines [28].

### 2.2. Input Features

For this study, we chose the pathology subject to the intervention and functional ability (mBI) before the intervention, with 24 additional variables extracted from the ADR, as input to the machine learning algorithms to assess functional ability at hospital discharge of the patient undergoing the intervention. The reason behind selecting only this group of features out of 120 data items in the original dataset is because many variables contained information that was not relevant to the task representing standard ADR voices such as the name of the hospital, the doctor in charge, type of facilities, etc. An overview of input variables is shown in Table 2. One of the main issues during the preparation of the input features was dealing with missing values as almost all variables were affected. The approach to address this issue was specific to the characteristics of each variable. For continuous variables such as mBI at admission and age, patients with missing values were excluded from the analysis due to the substantial influence of these variables on the outcome. Imputations were not considered suitable in this case. For categorical variables, the handling of missing values varied based on the variable’s nature. Proper attention was also given to outliers. From a statistical point of view, outliers are defined as data points that differ significantly from the rest of the data (i.e., having a negative mBI in admission) and can be very tricky to deal with to preserve the generalization of the machine learning model [29,30,31,32,33]. In this work, we defined outliers based on the mBI at admission and mBI at discharge, defining two normality conditions: (i) mBI at admission should be ≥10 according to standard rules [27], and (ii) mBI at discharge should be higher than that at admission. Cases where these conditions are not met are treated as outliers. The decision to keep or drop outliers was based on their nature. Cases in which (i) is not met are considered outliers since patients with mBI in admission < 10 are very rare and represent patients in very bad condition whose outcome cannot be correctly predicted and might alter the model to make bad predictions and lose generalization (ability to predict fair results in normal cases); for this reason, outliers of this nature are excluded. Meanwhile, cases in which (ii) is not met indicate that the rehabilitation was not successful, which is also a phenomenon that happens very rarely. However, the data is considered a good outlier since it may give important information regarding normal patient cases where the rehabilitation process does not give the desired results. Hence, these outliers kept improving the ability of the machine learning model to deal with special cases that rarely occur without significantly affecting the generalization of the model. Moreover, dummy variables and labels were created for categorical variables, to transform them into suitable input forms for machine learning algorithms. Meanwhile, continuous variables were scaled and normalized using the following formula:

(1) $$z=\frac{x-\mu }{\sigma }$$

where x represents the value of the continuous variable, μ is the mean of the continuous variable computed on the training set, σ is the standard deviation of the continuous variable computed from the training set, and z is the normalized value of x. An overview of the input features before preprocessing is shown in Table 2.

### 2.3. Outcome Variable

The variable that has served as a target for this study is the mBI after rehabilitation, which is a scale consisting of 10 items used to measure basic Activities of Daily Living. These 10 items pertain to tasks related to self-care and mobility, and each task is assigned a score reflecting the individual’s ability to perform it. A higher score indicates better performance ability, whereas a score of zero indicates a total inability to perform the task. The sum of individual scores for all 10 items ranges from a minimum of 0 (totally dependent) to a maximum of 100 (independent) [11]. Additionally, this instrument is easily administered by clinicians without requiring formal training or certification programs and has demonstrated good reliability [27].

### 2.4. Machine Learning Algorithms

To evaluate and compare machine learning algorithms, the dataset was split into two distinct subsets—one for NP and one for OP—and further partitioned into training and testing sets. The training set was generated by randomly selecting 80% of the dataset’s unique records, with the remaining 20% reserved for testing the algorithms. Five algorithms were utilized in the experiment, including four common algorithms (xGBT, LightGBM gradient boosting, and CatBoost [24,25,26]), as well as a custom algorithm developed by the researchers using stacking ensemble learning techniques to combine multiple machine learning models and form a more powerful one [34,35,36]. All models were developed using Python 3 and the scikit-learn [34] library for machine learning algorithms and statistical testing. Hyperparameters for each model were optimized using the grid search technique and 10-fold cross-validation solely on the training set. The way hyperparameter tuning is undertaken according to the grid search technique is straightforward. As input, it takes a specific model and set of values for each hyperparameter of that model generated from a linear space with predefined upper and lower bounds, and in order to find the best hyperparameters, it iteratively evaluates the model using cross-validation with different hyperparameter combinations from the set of values in input. At the end, the hyperparameters of the best-performing model on cross-validation are returned from the algorithm. This process aims to find the hyperparameters that give the best results for each model.

### 2.5. Customized Machine Learning Algorithm

**Figure 1:**
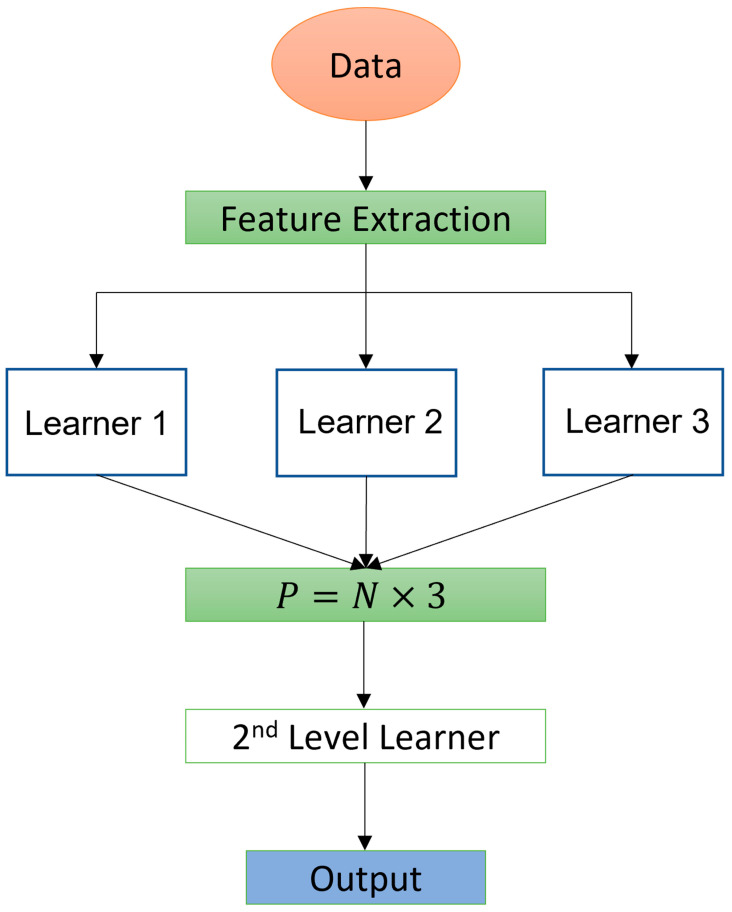
Illustration of the customized machine learning model.

**Figure 2:**
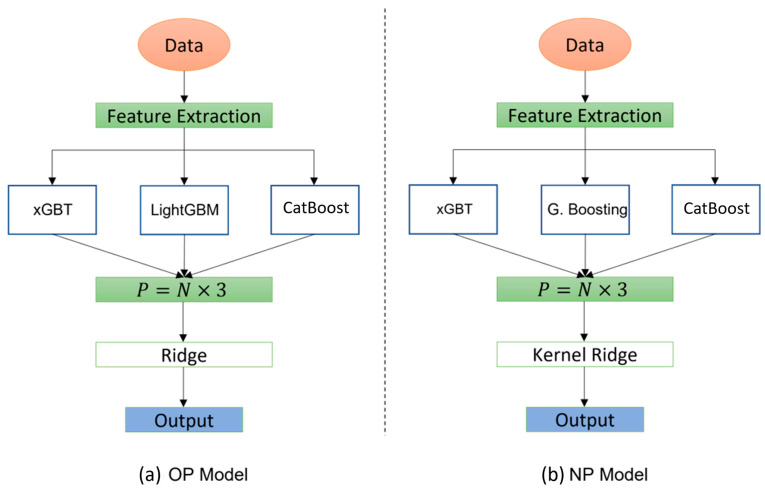
Illustration of customized models for OP (**a**) and NP (**b**).

The customized proposed method leverages all the selected features, using a joint architecture based on tree ensemble models such as CatBoost, gradient boosting, extreme gradient boosting, and light gradient boosting, and more simple models such as ridge regression, kernel ridge, and elastic net. The mentioned features are the result of the preprocessing phase applied to the data. The proposed system can be split into two levels. A first level, composed of powerful learners, takes as input the features that are a matrix of size n×m where n is the number of observations and m is the number of input features, and produces a vector $p_{i}$ of size n for each learner. Since multiple learners are present in this level, the overall output is a set of vectors $P=\left\{p_{1},p_{2},\ldots ,p_{d}\right\}$ and has a dimensionality n×d where d is the number of learners that compose this level of the model. The second level can also be called the meta learner, and it consists of a simple supervised model. In this level we used ridge regression and kernel ridge for the models dealing with OP and NP, respectively. At this point the model takes as input the produced matrix P from the first level and produces a vector of size n that represents the final prediction for each of the subtasks. The overall architecture can be seen as a stacked ensemble where learners of the first level are trained on the same data, but they come out with different properties, which is why we use a second level that tries to learn how to use these properties to create a more powerful and robust model. The following schema illustrates this ensemble (Figure 1). Regarding the first-layer learners, the tree ensembles were the models of choice due to their popularity and remarkable results obtained in regression tasks during the years. Mathematically speaking, these ensemble models can be defined as follows. For a given dataset with n samples and m features, $D=\left(x_{i},y_{i}\right)$ ($\left|D\right|=n,x_{i}\in R^{m},y_{i}\in R$), a tree ensemble model uses K additive functions to predict an outcome and the mathematical formulation of the outcome is as follows:

(2) $${\hat{y}}_{i}=\phi \left(x_{i}\right)=\sum_{k=1}^{K}f_{k}\left(x_{i}\right),$$

where $f_{k}$ represents the k−th additive function which corresponds to the k−th tree on the ensemble model. This mathematical formulation is generally adapted to all the ensemble trees while they differentiate from each other through the tree splitting algorithm [24,25,26]. Thus, based on this formula, the first predictions are produced directly from the features creating an n×l data matrix where l represents the number of learners which in our case is 3. Regarding the second-level learner, it is based on a simple ridge regression model that takes as input the produced n×l matrix and gives in output a vector of n elements optimized by minimizing the following formula:

(3) $$L=\sum_{i=i}^{n}{\left({\hat{y}}_{i}-y_{i}\right)}^{2}+\lambda \sum_{i=1}^{n}B_{i}^{2},$$

where ${\hat{y}}_{i}$ is the estimated value, $y_{i}$ is the ground truth, $B_{i}^{2}$ is the penalization term, and L stands for loss. Thus, essentially, ridge regression is nothing more than a residual sum of squares (RSS) plus a squared penalization term. In addition, since we have two different macro groups of patients, NP and OP, the customized models for each have different configurations which means a different learner in the first and second layers is chosen based on performance with respect to the metric of interest. An illustration of the models for OP and NP is shown in Figure 2a,b, respectively. As can be seen, there are some differences on the used models in the first and second layers. In the first layer for OP, LightGBM is used in combination with xGBT and CatBoost, whereas for NP we used Gradient Boosting instead of LightGBM. In the second layer, for OP we used the classic ridge regression whereas for NP the kernelized version of it is used. This is same as kernel ridge with the addition of the kernel trick to create non-linearity.

### 2.6. Outcome Statistical Analysis

The machine learning prediction algorithms, which were trained using the training cohort, were evaluated on the testing cohort by measuring the RMSE, or root mean squared error. This metric represents the absolute fit of the model to the data by calculating the square root of the residuals’ variance [35]. The choice to use RMSE was based on the nature of the problem at hand, as we were interested in precisely gauging the model’s predictive capabilities. To further support the findings with statistical evidence, we also calculated the R-squared value, a statistical measure that indicates the proportion of variance in the dependent variable explained by independent variables in the regression model [34]. Additionally, a 95% confidence interval and *p*-values were calculated using a one-sample, two-tailed *t*-test.

## 3. Results

### 3.1. Data Extraction

**Figure 3:**
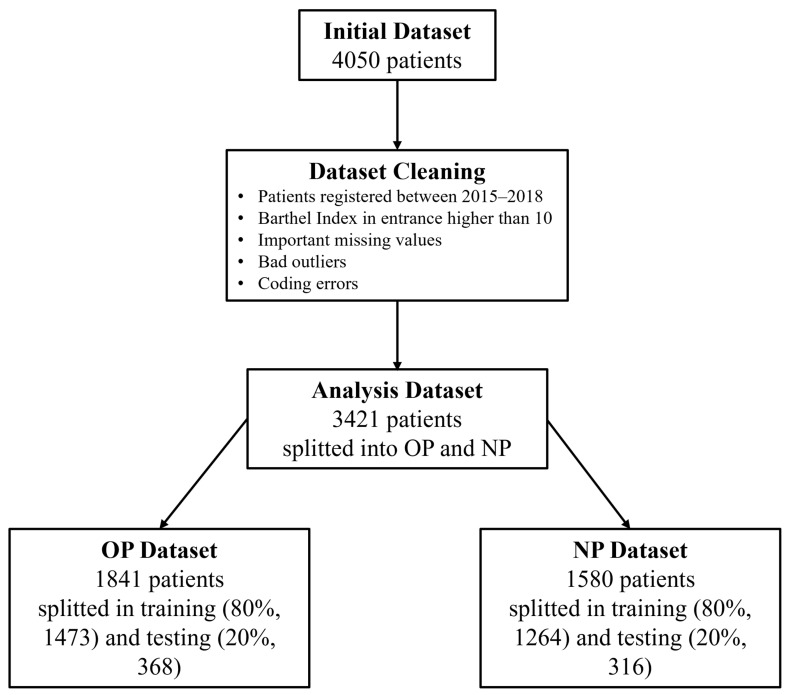
Illustration of data cleaning and splitting process.

The dataset provided for this study contained a total of 4050 records, each corresponding to a unique patient. After undergoing cleaning and preprocessing, the number of remaining patients was reduced to 3421. Various factors led to exclusions, such as coding errors, missing values, and outliers that were deemed inappropriate for the scope of this research. After the cleaning process was completed, the dataset was divided into two groups based on ICD9-CM codes corresponding to the patients’ base rehabilitation pathology: OP patients (1841) and NP patients (1580). Each group was further subdivided into a training set (80% of patients) and a testing set (20% of patients), resulting in 1473 training patients for OP and 1264 training patients for NP, along with 368 testing patients for OP and 316 testing patients for NP. A diagram of the data extraction process can be found in Figure 3.

### 3.2. Variable Importance Ranking

To gain a better understanding of how input features impact the machine learning models, we conducted a variable importance analysis. The aim was to assign an importance score to each variable based on its influence on the final outcome, and the analysis was performed separately for the xGBT, LightGBM, and CatBoost models [37]. We used inbuilt functions from the scikit-learn library to rank the variables based on the amount of variance reduction that each variable caused to the final model output, as described in [38]. We conducted ranking separately for OP and NP cases, and Table 3 and Table 4 present the top 10 important features for each model for OP and NP, respectively. Based on the results shown in Table 3 and Table 4, the standard important features in the top 10 ranking are Barthel Index at admission, Age, Behavior impairment, Cognitive impairment, and the “no associated pathologies” variable in all the machine learning models. Notably, each model provides almost the same results in both OP and NP cases, with differences in the base pathology category, which defines the base pathology and indicates whether the patient is part of NP and OP according to the ICD9-CM classification. CatBoost and XGB have very similar results with minor changes, whereas LightGBM is very different compared with them.

### 3.3. Prediction Accuracy and Analysis

**Figure 4:**
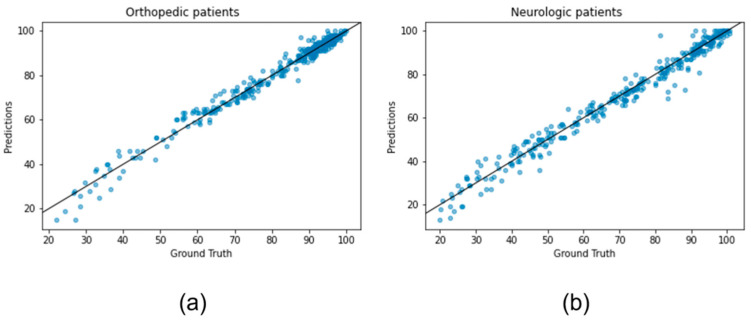
Comparison between the ground truth distribution and the predicted distribution of mBI at discharge for OP (**a**) and NP (**b**).

Table 5 and Table 6 display the prediction accuracy based on RMSE and the goodness of fit explained by R-squared for all models in OP and NP. The results indicate that all models perform remarkably well [22] in both metrics, with the customized model achieving slightly lower RMSE for both OP and NP (6.58 and 8.66, respectively). In terms of R-squared, LightGBM performs the best for OP data (0.868), whereas xGBT is the top performer for NP data (0.85). The reason why the customized model gives better results in terms of RMSE but not in terms of R-squared (even though the difference is very small considering the scale) is because the customized model is slightly more biased in proportion to RMSE than the other models, as can be seen in Table 5 and Table 6. In this case, the bias indicates that the model is giving higher importance to some variables. However, as long as R-squared is significant, the metric of interest for our purpose is RMSE because it shows the absolute mean error of the prediction and it is also on the same scale as the dependent variable [35], in our case mBI at discharge. In addition, in Figure 4a a simple comparison between estimated mBI and ground truth mBI is shown through scatter plots for both NP and OP datasets with the x axis representing the ground truth and the y axis representing the predictions. As can be seen from the plots, the points are distributed mostly close to the x=y line except for some outlier points. Thus, the results of our model are meaningful, and the prediction distribution is close to the real one. Moreover, to further illustrate the usefulness of the model, in addition to the scatterplot, we demonstrate the Bland–Altman plot in Figure 4 which also shows a comparison between ground truth and the predicted variable in terms of means and differences. From the plots, we can see that for both cases the difference mean is close to 0 (−0.63 for NP and −0.25 for OP, respectively) which again proves the distributions of predictions and ground truth are similar. Furthermore, from the distribution of points in the plot we can observe that the model is not consistently overestimating or underestimating the outcome, and most of the points fall inside the 95% interval bounded from 1.96±std where the std shows the standard deviation of the differences between ground truth and predictions. To make our results statistically more significant, in Table 5 and Table 6 we report the 95% confidence intervals and *p*-values for all models. As can be seen, our customized model has the smallest interval among all for both NP and OP. Regarding the *p*-value, it was calculated by performing the one-sample two-tailed t. In essence, the one-sample two-tailed *t*-test is used to determine whether two distributions are significantly different from each other, in our case the prediction distribution and the ground truth distribution of the test sample, based on the means of the samples. In other words, we are testing the hypothesis that the mean of the prediction distribution is equal to the mean of the ground truth data. To do so, we first extract the prediction distribution from the test set by applying the models, and afterward use this distribution together with the mean of the ground truth test set to run the one-sample two-tailed t-test and extract the *p*-value with respect to the hypothesis. As can be observed, the *p*-value for our model is 0.84 in the case of NP and 0.85 for OP, showing with a high probability that there is not enough evidence to throw the null hypothesis, meaning that there is no statistical evidence to deny the equality between the mean of the prediction’s distribution and the ground truth distribution.

### 3.4. Ablation Study

A crucial aspect of our study was to determine the optimal set of algorithms to develop a robust predictive model. We conducted a series of experiments using various algorithms and ensemble techniques on both OP and NP datasets. The primary aim of these experiments was to identify the top three learning algorithms for the first layer and determine the best approach for the second layer (if necessary) of the ensemble. We initially selected a group of pre-existing algorithms such as xGBT, LightGBM, Gradient Boosting, and Support Vector Regressor (SVR) for the first layer. For the second layer, we chose a group of simple learning algorithms, including lasso, ridge regression, kernel ridge regression, elastic net, and a manual weighting approach where each of the first layer algorithms was assigned an equal weight of 0.33. Table 7 displays the results obtained during the experiments for all the various algorithm combinations we tried. We measured the RMSE using cross-validation on the training set for each combination to extract the best parameters and then validated each model on the test set. Our findings indicate that some of the models perform well on their own without the addition of a second layer. However, the best results are achieved in combination with a second layer in both cases. Specifically, in the case of NP, the combined model of the first layer consisting of xGBT, CatBoost, and G. Boosting independently produced RMSE values of 8.9, 9.09, and 9.18, respectively. However, with the addition of a second layer to combine them, the performance improved to an RMSE of 8.85 in the case of Ridge and 8.66 in the case of Kernel Ridge. In contrast, for OP, the best-performing first-layer models, consisting of xGBT, LightGBM, and CatBoost, produced RMSE values of 6.71, 6.59, and 6.8, respectively. The addition of a second layer slightly improved the performance, with an RMSE of 6.58 in the case of Ridge. Overall, our results suggest that the second layer is more beneficial for NP patients than for OP patients, providing a higher performance improvement in terms of RMSE.

## 4. Discussion

**Figure 5:**
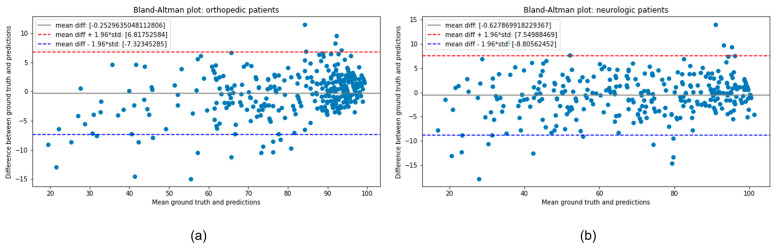
Bland–Altman plots on OP (**a**) and NP (**b**). std: standard deviation.

This study aimed to identify an algorithm that, using data extracted from ADR, was able to provide a prediction of mBI at discharge. For this purpose, the dataset was split into two independent sections based on the pathology object of the rehabilitation program: neurological and orthopedics patients. The results of the customized predictive model showed RMSE equal to 6.58 and 8.66 in orthopedics and neurological patients, respectively. These results show that it performs similarly to the three traditional machine learning models, namely CatBoost, xGBT, and LightGBM. More specifically, CatBoost gives an absolute error of 6.8 for OP and 9.09 for NP, xGBT gives 6.71 for OP and 8.9 for NP, LightGBM gives 6.59 for OP and 9.23 for NP, and as noticed all of them produce a slightly higher absolute mean error compared with a customized model which is more noticeable in the case of NP. From a clinical point of view, according to the clinicians’ part of this study, the obtained data can be considered valid support to predict an adequate rehabilitation prognosis right from the patient’s admission to a post-acute rehabilitation department [39]. To support the conclusion, we compare the RMSE results with the standard deviation that exists in the target variable. Specifically, the standard deviation is 22.71 and 17.88 for NP and OP, respectively, which means the approach can be useful in practice from a clinical point of view with RMSE being much lower than the standard deviation. Moreover, regarding the obtained RMSE or the absolute mean error values, they could be explained based on the quality of the used dataset, which could be affected by the subjectivity of the operator who fills in the single form. On the other hand, to be noted is the fact that the models perform better for OP data than NP data in both the RMSE and R-squared metrics. This difference for neurological patients could be explained by the clinical and therefore functional variability (which can also be noted from the standard deviation) that distinguishes this category of patients: it is in fact known that, since the diagnosis of stroke or multiple sclerosis, the variability among cases can be considerably different [40]. This obviously impacts functionality and therefore the rehabilitation process, and consequently has effects on the outcome and the possibility of predicting the trend in an optimal way instead for patients with disabilities of orthopedic origin (in this case, the patients examined were almost all outcomes of hip replacement surgery). Furthermore, differences found between the two groups could be due to individual variability in terms of the appearance of any complications occurring during the rehabilitation period, responsible for changes in the patient’s clinical status and thus determining unpredictable changes on Barthel output (Figure 4 and Figure 5).

### 4.1. Study Strengths and Limitations

In this study, the actual mBI score at discharge could be predicted with high accuracy; thus, the approach can truly assist the clinical practice in rehabilitation wards. The current study’s use of multiple machine learning algorithms has suggested intriguing variations in the importance of different variables depending on the modeling technique and the task. The explored models were based on decision trees [38]. They closely resembled each other, especially in the case of CatBoost and XGB, where the top 10 important features were almost the same, presenting some minor differences in variable ranking. Finally, this study shows that machine learning can have an important impact on the development of intelligent tools that can help medical improvement and can also serve as a breakthrough to a new method of applying machine learning in rehabilitation process enhancement. Limitations we faced during this study are related to data quality, data quantity [41,42], and machine learning. Firstly, since machine learning models are data-centric models, the quality of data is particularly important. In our case, the main problem regarding data quality is found with the most important input feature (based on the feature importance ranking of all the algorithms) and with the outcome variable. This is due to the nature of mBI and the way it is measured, which is characterized by a high degree of subjectivity. Secondly, the quantity of data being used is very important. It is said that machine learning models learn from “experience” which comes with the data. This fact is directly related to the quantity of data that machine learning is taking as input during training; the higher the quantity, the better it is. In our case, the quantity was very low both for NP and OP cases. The type of therapy was not factored into the algorithm. Having it as part of future algorithms would be beneficial for therapy progression in the future. The categories are very broad and should be limited in future works. The type of rehabilitation activities that feed into the narrower diagnosis-based algorithm will allow for a more useful tool for therapists in the future. Finally, it is acknowledged that machine learning models act like a black box which makes their interpretation extremely difficult. This interpretation difficulty directly affects the understanding of the relationship that exists between input variables and the outcome.

### 4.2. Machine Learning Feasibility and Implications

The possibilities and implications of machine learning extend far beyond the scope of our study. Machine learning systems offer a powerful tool that could enhance the rehabilitation process by facilitating the design of personalized treatment plans and enabling more efficient and accurate monitoring of patients. Machine learning systems excel at analyzing data, and as more data becomes available, these systems become even more proficient at performing multiple tasks.

### 4.3. Limitations

There are several implications to consider. Firstly, the lack of standardization in rehabilitation makes it difficult to develop unique tools according to well-defined standards. This is primarily due to the absence of standardized data, which is a challenge not only in rehabilitation but in the healthcare industry, given the diverse range of protocols, techniques, technologies, routines, and so on used in different healthcare units. Secondly, biased data is a significant concern in applying machine learning to rehabilitation, as human evaluation can influence the data acquisition process. For example, mBI used in our study is subject to bias when expert clinicians evaluate a patient’s functional ability based only on observations and medical records. Even highly qualified clinicians cannot guarantee an unbiased quantitative evaluation of mBI, especially when multiple clinicians are involved.

The type of therapy was not considered in the algorithm. Having that as part of future algorithms would be beneficial for therapy progression in the future. The categories are very broad and should be limited in future work. The type of rehabilitation activities that feed into the narrower diagnosis-based algorithm will allow for a more useful tool for therapists in the future. Finally, using machine learning in rehabilitation raises ethical and privacy concerns, as collecting and using personal health data requires careful management to ensure patient confidentiality and informed consent. Furthermore, relying on technology may diminish human interaction and empathy, which are essential aspects of the rehabilitation process. Therefore, although the feasibility of using machine learning in rehabilitation is promising, it must be approached with caution and sensitivity to ensure that patients receive the best possible care.

## 5. Conclusions

In this work, an original combination of machine learning models and careful data preprocessing is used to realize an original method for predicting effectiveness in rehabilitation. Obtained results showed how all the proposed models, including the customized one, perform significantly well, thus demonstrating how the use of machine learning can help in predicting mBI and improving the rehabilitation process accurately. In conclusion, an enhancement of the dataset, which should be extended by integrating new measures, is needed. Furthermore, an increase in the sample size and quality of data improvement, which showed contradictory elements probably due to the human factor, will make model learning more effective. As for future development, interesting topics concern the investigation of new models, deep learning models, and also other feature engineering approaches that could help in extracting more useful information.

## Figures and Tables

**Table 1:** Demographic insights on patients.

Demographic Data	Neurology Patients	Orthopedic Patients
Number of patients	1580	1841
Mean age	69±13	72±11
Mean mBI at admission	31±13	37±10
Mean mBI at discharge	72±23	81±18
Mean mBI change	41±18	44±14
Mean length of hospitalization	45±14	30±9
Gender: Male%	53.5%	34.1%
Gender: Female%	46.5%	65.9%
Nationality: Italian%	98.1%	99.2%
Nationality: Other%	1.9%	0.8%

**Table 2:** Variables used as input to machine learning algorithms. The ranges 710–739 and 320–389 show the macro-categories for orthopedic and neurologic classification based on ICF9-CM associated with base pathology or COD_27 and used to divide patients into OP and MP.

ADR Code	Variable Name	Type	Possible Values
COD_52	mBI at admission	Quantitative	[0–100]
Age	Age	Quantitative	[18–97]
Gender	Gender	Categorical	{0 (M), 1 (F)}
COD_26	Pathology subject to rehabilitation	Categorical	ICD9-CM
COD_27	Base pathology associated to intervention	Categorical	ICD9-CM [710–739, 320–389]
COD_28-35	Associated pathologies	Categorical	ICDM9-CM
COD_36	Cognitive impairment	Categorical	[0 (N), 1 (Y)]
COD_37	Behavior impairment	Categorical	[0 (N), 1 (Y)]
COD_38	Communication/Language impairment	Categorical	[0 (N), 1 (Y)]
COD_39	Sensory impairment	Categorical	[0 (N), 1 (Y)]
COD_40	Manipulation impairment	Categorical	[0 (N), 1 (Y)]
COD_41	Balance impairment	Categorical	[0 (N), 1 (Y)]
COD_42	Locomotion impairment	Categorical	[0 (N), 1 (Y)]
COD_43	Cardiovascular impairment	Categorical	[0 (N), 1 (Y)]
COD_44	Respiratory system impairment	Categorical	[0 (N), 1 (Y)]
COD_45	Ulcer	Categorical	[0 (N), 1 (Y)]
COD_46	Sphincter control impairment	Categorical	[0 (N), 1 (Y)]
COD_47	Urinary system impairment	Categorical	[0 (N), 1 (Y)]
COD_48	Nutrition impairment	Categorical	[0 (N), 1 (Y)]

**Table 3:** Top 10 important features from the three machine learning algorithms, CatBoost, LightGBM, and XGB on OP patients.

CatBoost	LightGBM	XGB
mBI at admission	mBI at admission	mBI at admission
Age	Cognitive impairment	No associated pathologies
No associated pathologies	Ulcer impairment	Age
Handling impairment	Age	Handling impairment
Behavior impairment	Amputees	Cognitive impairments
Cognitive impairments	Organ or tissue replaced by other means	Behavior impairment
Nutrition impairment	Urinary impairment	Nutrition impairment
Hypertension	No associated pathologies	Communication impairment
Vertebral pathology	Femur osteosynthesis	Gender
Control impairment	Behavior impairment	Hypertension

**Table 4:** Top 10 important features from the three machine learning algorithms, CatBoost, LightGBM, and XGB on NP patients.

CatBoost	LightGBM	XGB
mBI at admission	mBI at admission	mBI at admission
Age	Cognitive impairment	No associated pathologies
No associated pathologies	Ulcers impairment	Age
Handling impairment	Age	Handling impairment
Behavior impairment	Parkinson	Cognitive impairment
Cognitive impairment	Organ or tissue replaced by other means	Behavior impairment
Nutrition impairment	Urinary impairment	Nutrition impairment
Hypertension	No associated pathologies	Communication impairment
Parkinson	Non-traumatic myelo-radiculopathies	Gender
Sphincter control impairment	Behavior impairments	Hypertension

**Table 5:** Root mean square error (RMSE), bias, confidence interval (CI), *p*-value, and R-squared statistics for all models for OP.

Model	RMSE for OP	Bias	R-Squared	CI (95%)	*p*-Value	RMSE CV
xGBT	6.71	4.42	0.862	78.15–83.1	0.77	6.01
LightGBM	6.59	4.41	0.868	79.1–82.9	0.79	5.95
CatBoost	6.8	4.62	0.84	78.6–83.27	0.76	6.21
Our model	6.58	4.5	0.837	79.21–82.92	0.79	5.91

**Table 6:** Root mean squared error (RMSE), bias, R-squared, confidence interval (CI), and *p*-value for all models for NP.

Model	RMSE for NP	Bias	R-Squared	CI (95%)	*p*-Value	RMSE CV
xGBT	8.9	5.78	0.85	67.81–73.98	0.79	7.11
LightGBM	9.23	5.99	0.835	66.8–73.07	0.61	8.02
CatBoost	9.09	5.92	0.84	67.79–73.1	0.72	8.1
Our model	8.66	5.81	0.836	67.78–72.71	0.81	6.87

**Table 7:** Ablation study for choosing best model combinations for both NP and OP according to RMSE metric.

First Layer	Second Layer	RMSE NP	RMSE OP
xGBT	-	8.9	6.71
LightGBM	-	9.23	6.59
CatBoost	-	9.09	6.8
G. Boosting	-	9.18	6.91
SVR	-	11.2	9.17
xGBT + CatBoost + LightGBM	Weighting	9.32	6.77
xGBT + CatBoost + G. Boosting	Weighting	9.2	6.84
xGBT + CatBoost + SVR	Weighting	10.1	8.21
xGBT + CatBoost + LightGBM	Lasso	9.28	6.78
xGBT + CatBoost + G. Boosting	Lasso	9.11	6.81
xGBT + CatBoost + SVR	Lasso	9.91	8.14
xGBT + CatBoost + LightGBM	Ridge	9.01	6.58
xGBT + CatBoost + G. Boosting	Ridge	8.85	6.77
xGBT + CatBoost + SVR	Ridge	9.42	8.21
xGBT + CatBoost + LightGBM	Kernel Ridge	8.87	6.59
xGBT + CatBoost + G. Boosting	Kernel Ridge	8.66	6.69
xGBT + CatBoost + SVR	Kernel Ridge	9.21	7.92

## Data Availability

Data is contained within the article.
